# Prevalence of Chronic Kidney Disease and Associated Factors Among Adult HIV/AIDS Patients on HAART in Hiwot Fana Comprehensive Specialized Hospital, Eastern Ethiopia: A Cross‐Sectional Study

**DOI:** 10.1002/hsr2.71232

**Published:** 2025-09-12

**Authors:** Gada Diba, Ahmedmenewer Abdu, Ephrem Tefera Solomon, Winner Kucha

**Affiliations:** ^1^ School of Medical Laboratory Sciences, College of Health and Medical Sciences Haramaya University Harar Ethiopia

**Keywords:** associated factor, chronic kidney disease, highly active antiretroviral therapy, prevalence

## Abstract

**Background and Aims:**

Chronic kidney disease related to human immunodeficiency virus infection has become a significant concern in sub‐Saharan Africa, primarily due to the high prevalence of the virus, along with delays in diagnosis and the initiation of highly active antiretroviral therapy. This study aimed to assess the prevalence of chronic kidney disease and its associated factors among human immunodeficiency virus patients receiving highly active antiretroviral therapy in Hiwot Fana Comprehensive Specialized Hospital, Harar, Eastern Ethiopia, from November 20, 2023, to February 22, 2024.

**Methods:**

A hospital‐based cross‐sectional study was carried out, and 228 study participants were enrolled using a convenient sampling technique. Trained laboratory professional and nurse utilized semi‐structured questionnaires to gather sociodemographic, clinical, and lifestyle data. The collected data were entered into EpiData version 4.6 and then exported to SPSS version 27 for analysis. Bivariate and multivariable logistic regression analyses were used to assess factors associated with chronic kidney disease. Statistical significance was determined by a *p*‐value less than 0.05.

**Results:**

The overall prevalence of chronic kidney disease among study participants was 17.5% (40/228) (95% CI: 12.8%–23.1%). Older age [AOR: 3.6, 95% CI: 1.08–11.96, *p* = 0.036] and family history of kidney disease [AOR: 2.9, 95% CI: 1.27–7.04, *p* = 0.012] were significantly associated with chronic kidney disease.

**Conclusion:**

Regular monitoring of kidney function is important for older individuals and those with a family history of kidney disease, to promptly identify and effectively manage chronic kidney disease in individuals on highly active antiretroviral therapy.

**Abbreviations**
CKD: chronic kidney disease
CKD‐EPI: Chronic Kidney Disease Epidemiology Collaboration
eGFR: estimated glomerular filtration rate
HAART: highly active antiretroviral therapy
HFCSH: Hiwot Fana Comprehensive Specialized Hospital
HIV: human immunodeficiency virus
HTN: hypertension
MDRD: modification of diet in renal diseases
PLWHA: people living with HIV/AIDS

## 1 Background

Kidney disease refers to conditions that damage or impair the function of the kidneys [1]. Based on duration, the disease can be classified into two categories (acute and chronic). Acute kidney disease (acute kidney injury) refers to a sudden and often reversible loss of kidney function. It typically occurs over a short period, ranging from hours to days [2]. Chronic kidney disease (CKD) is a medical condition marked by a gradual, irreversible, and persistent decrease in renal function over a period of months to years, which impairs their ability to effectively filter waste and perform other essential functions [2, 3].

CKD is a serious global public health issue. The prevalence of CKD is rapidly rising, primarily due to escalating numbers of risk factors and the growing burden of non‐communicable disease. According to estimates, CKD affects a substantial proportion of the world population, ranging from 8% to 16%, with particularly high prevalence observed in low‐ to middle‐income countries (LMICs) [4].

Globally, it is estimated that 6.4% of people living with HIV/AIDS (PLWHA) have CKD. This prevalence, which varies by area, affects 3.7% of the population in Europe, 5.7% in Asia, 7.1% in North America, and 7.9% in Africa [5]. As a result of the high prevalence of CKD, along with the late diagnosis of human immunodeficiency virus (HIV) and delays in starting highly active antiretroviral therapy (HAART), CKD has received increased attention in Africa [6]. With the administration of HAART, the prevalence of HIV‐associated nephropathy decreased, but there is still a nearly fourfold higher risk of kidney disease, including CKD, in the PLWH compared to the general population [7].

CKD can be identified through various diagnostic measures, including glomerular filtration rate (GFR), proteinuria, imaging techniques, and renal biopsy [8]. Estimating GFR can be achieved using different equations, including Modification of Diet in Renal Disease (MDRD) equation, Chronic Kidney Disease Epidemiology Collaboration (CKD‐EPI) equation, the Cockcroft‐Gault equation, and the Bedside Schwartz equation. Among these, the MDRD and CKD‐EPI equations are commonly used in adults. The CKD‐EPI equation provides a more accurate estimation of GFR compared to MDRD and is widely accepted in clinical practice [9]. Nowadays, the concept of machine learning model has been introduced for predicting estimated GFR (eGFR) and staging of CKD, including methods such as Support Vector Machine, Decision Trees, Random Forest, and XGBoost [10].

Previous studies primarily classified CKD based on eGFR criteria alone [11, 12]. Additionally, some studies utilized the less accurate Cockcroft‐Gault formula for estimating GFR [13, 14, 15, 16]. These studies have highlighted the need for future research to address these gaps. Furthermore, there is limited data regarding CKD and its associated factors among adult HIV patients receiving HAART in Ethiopia, specifically in Harar. Therefore, the purpose of this study was to determine the prevalence of CKD and its associated factors among adult HIV patients receiving HAART at Hiwot Fana Comprehensive Specialized Hospital (HFCSH).

## 2 Materials and Methods

### 2.1 Study Design, Study Area, and Period

A hospital‐based cross‐sectional study was conducted in the Harari Region at HFCSH from November 20, 2023, to February 22, 2024.

### 2.2 Population

#### 2.2.1 Source Population

All HIV/AIDS patients at HFCSH antiretroviral therapy center receiving HAART.

#### 2.2.2 Study Population

All adult HIV/AIDS patients at HFCSH antiretroviral therapy center who were receiving HAART during the study period and met the inclusion criteria.

### 2.3 Inclusion and Exclusion Criteria

#### 2.3.1 Inclusion Criteria

Adults aged 15 years and above who were HIV patients receiving HAART for more than 6 months and visited HFCSH during the study period.

#### 2.3.2 Exclusion Criteria

The study excluded seriously ill patients who were unable to provide responses, as well as those diagnosed with diabetes or hypertension, since these conditions are known risk factors for CKD and could lead to a higher prevalence of CKD among HIV patients. Additionally, individuals with incomplete clinical and laboratory data were excluded to ensure the integrity of the study findings. Pregnant women were also excluded from the study.

### 2.4 Operational Definition

CKD was defined as either an eGFR below 60 mL/min/1.73 m^2^ or the presence of persistent proteinuria lasting for more than 3 months when the eGFR is equal to or greater than 60 mL/min/1.73 m^2^ [17].

### 2.5 Sample Size Determination and Sampling Technique/Procedure

**Figure 1:**
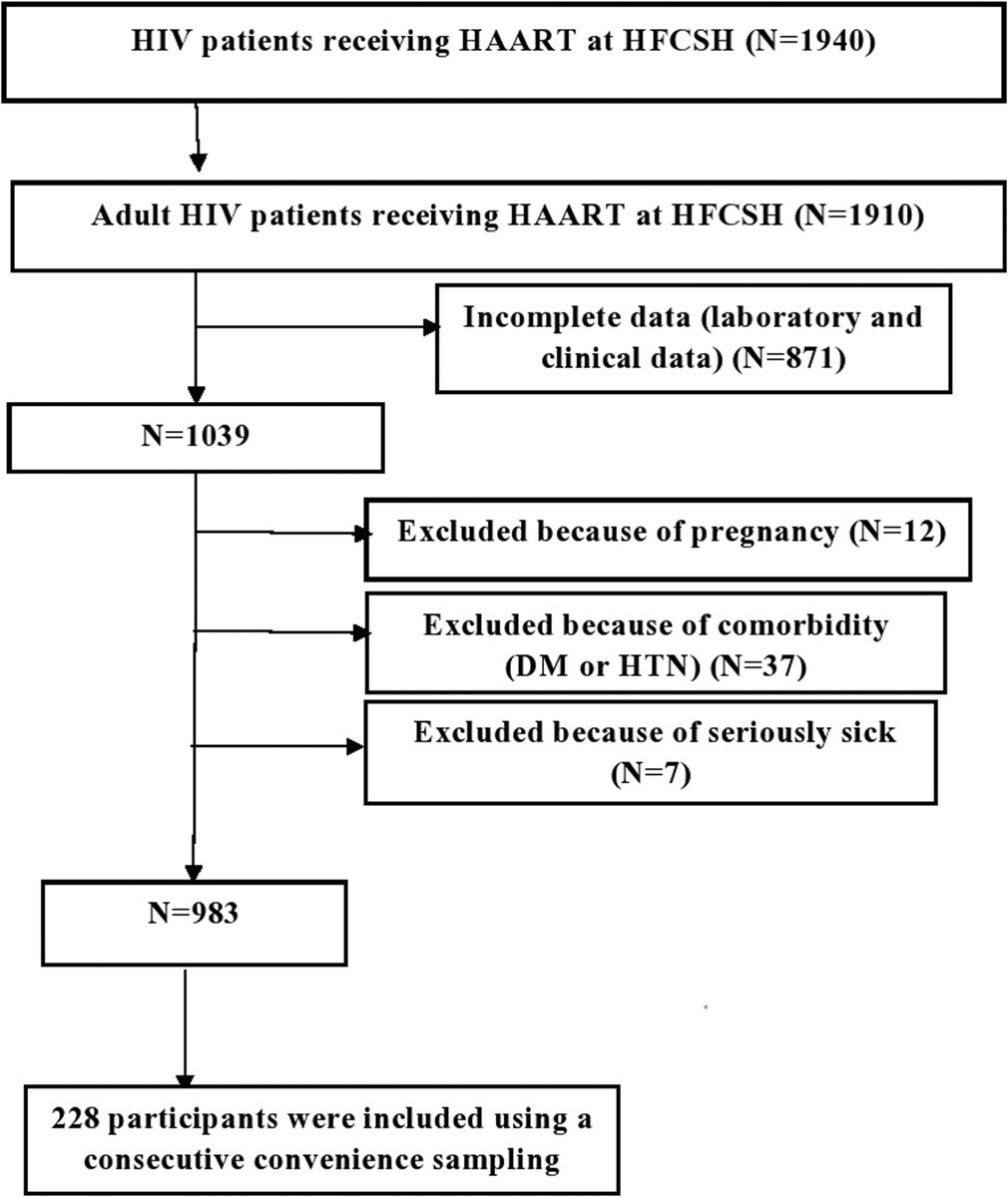
Schematic representation of the sampling procedure for adult HIV patients on HAART at HFCSH, 2024. DM, diabetes mellitus; HAART, highly active antiretroviral therapy; HFCSH, Hiwot Fana Comprehensive Specialized Hospital; HTN, hypertension.

The sample size for this study was determined using the single population proportion formula (*n* = (Zα/2)^2^ p (1−p)/d^2^) to estimate a single population proportion with a 95% confidence interval (CI). Assuming a margin of error of 5% and a non‐response rate of 10%, the value of Zα/2 (corresponding to a 95% confidence level) is 1.96. Based on a previous study conducted at the University of Gondar Referral Hospital in Gondar, Ethiopia, the prevalence of CKD among HIV patients on HAART was reported as 16.1% [6]. A total sample size of 228 was obtained through a convenient sampling method (Figure 1).

### 2.6 Data Collection Methods

Data collection was carried out by trained nurse and laboratory professional using a semi‐structured questionnaire that was derived from different sources [6, 18, 19, 20]. The questionnaire was initially developed in English and subsequently translated into local languages (Afan Oromo and Amharic). To ensure consistency, the translated versions were back‐translated to English. The data were collected by one nurse and one medical laboratory professional, who were supervised by a senior medical laboratory sciences professional. Before the actual data collection, the data collectors received training on how to collect data from study participants.

### 2.7 Sample Collection, Processing, Handling, and Laboratory Methods

Study participants were asked to give the required sample. Approximately 10 mL of urine was collected using a clean, leak‐proof, and dry urine cup for assessing proteinuria. Additionally, 5 mL of venous blood was collected using a serum separator tube to determine serum creatinine levels. For creatinine determination, a serum sample was immediately separated by allowing the whole blood to sit on a cloth for 30 min. It was then centrifuged at 3000 rpm for 10 min. A serum creatinine level was determined using Cobas C311 analyzer (Roche Diagnostics International Ltd., Rotkreuz, Switzerland) based on the kinetic Jaffe reaction and reported in mg/dL. To ensure accuracy, daily quality control checks and regular calibration of the instruments were performed. Urine specimens were tested for protein levels immediately after sample collection using urine dipstick tests (Shanghai SNWI Co Ltd., Shanghai, China). The results for urine protein level were reported semi‐quantitatively as negative, +1, +2, +3, or +4. Both serum creatinine and proteinuria were assessed for the study participants, and the diagnosis of CKD was based on the respective values obtained. The eGFR was calculated using the CKD‐EPI 2021 equation, which is considered more accurate than the previous MDRD equation for non‐pregnant adults, particularly in estimating GFR at higher levels [21].

### 2.8 Methods of Data Analysis

**Figure 2:**
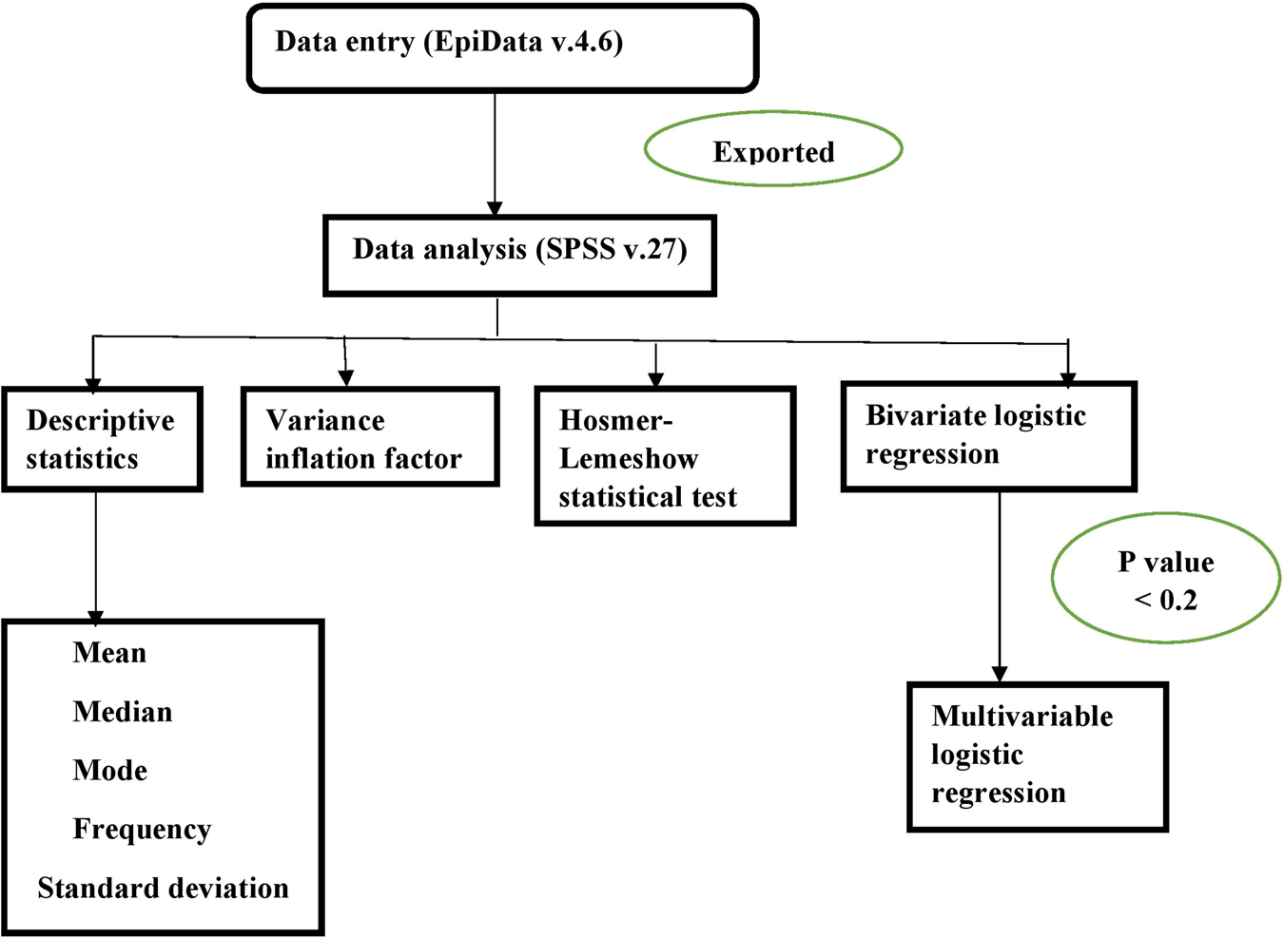
Flowchart of statistical analysis steps for adult HIV patients on HAART at HFCSH, 2024.

After checking for completeness and consistency of the collected data, the data were entered into EpiData version 4.6 and then exported to SPSS version 27 for analysis. Descriptive statistical analysis was employed to provide an overview of the socio‐demographic, clinical, and lifestyle characteristics of study participants, as well as the prevalence of CKD. The findings were presented using frequencies, percentages, tables, and figures. To assess the presence of multicollinearity among the independent variables, the variance inflation factor (VIF) was examined, and no variables were found to have a VIF exceeding 1.9. The goodness of fit of the model was checked using Hosmer‐Lemeshow statistical test. Bivariate logistic regression was used to assess the crude association between independent and dependent variables. Independent variables with a *p‐*value of ≤ 0.20 in the bivariate analysis were considered for inclusion in the multivariable logistic regression model to control for potential confounding variables. Finally, multivariable logistic regression was employed to identify factors independently associated with CKD, with statistical significance determined by a *p‐*value < 0.05 (Figure 2).

### 2.9 Data Quality Control

During the pre‐analytical phase, training was provided for data collectors, and detailed data collection tools were developed. Pre‐test was conducted on 5% of study participants in Jugal Hospital to identify problems with the data collection instrument and find possible solutions, and the raw data were checked every day for completeness and consistency of filling the questionnaire during and after data collection (before entry). Data cleaning and validation procedures were conducted to identify and rectify any inconsistencies or outliers. Standard operating procedures were followed for specimen collection and handling. Quality control materials were used to monitor the performance and accuracy of laboratory tests.

### 2.10 Ethical Considerations

Ethical clearance was obtained from Institutional Health Research Ethical Review Committee (Ref No: IHRERC/215/2023) belonging to College of Health and Medical Sciences, Haramaya University. The permission letter was taken from the clinical director of HFCSH. Written informed consent was obtained from each study participant after providing a detailed explanation of the research, including its aim, procedures, period, possible risks, and benefits. Every patient had the right to decide whether to participate in the study, and no coercion was imposed on those who declined participation.

## 3 Results

### 3.1 Socio‐Demographic Characteristics

The study included a total of 228 HIV patients who were receiving HAART. Among them, 69.7% (159/228) were females, and 30.3% (69/228) were males. Eighty‐six percent (196/228) of the study participants were living in urban areas, and 40.3% (92/228) were within the age group of 40–49 years. The mean (standard deviation) age of the study participants was 41.8 (10.2 years) (Table 1).

**Table 1:** Sociodemographic characteristics of HIV patients on HAART at HFCSH, Harar, Eastern Ethiopia, 2024 (*n* = 228).

Variables	Categories	Frequency (%)
Age (mean ± SD)	41.8 ± 10.2 years	
	15–29	33 (14.5)
	30–39	49 (21.5)
	40–49	92 (40.3)
	≥ 50	54 (23.7)
Sex	Male	69 (30.3)
	Female	159 (69.7)
Residence	Urban	196 (86.0)
	Rural	32 (14.0)
Religion	Protestant	23 (10.1)
	Orthodox	148 (64.9)
	Muslim	55 (24.1)
	Other	2 (0.9)
Ethnicity	Oromo	89 (39.0)
	Amhara	119 (52.2)
	Harari	9 (4.0)
	Other	11 (4.8)
Education status	No formal education	29 (12.7)
	Primary education	110 (48.3)
	Secondary education	60 (26.3)
	College and above	29 (12.7)
Occupational status	Government employee	67 (29.4)
	Private employee	75 (32.9)
	Housewife	60 (26.3)
	Student	15 (6.6)
	Other	11 (4.8)
Marital status	Single	35 (15.3)
	Married	120 (52.6)
	Divorced	43 (18.9)
	Widowed	30 (13.2)
Monthly income	< 3000	131 (57.5)
	3000–6000	66 (28.9)
	> 6000	31 (13.6)

### 3.2 Lifestyle Characteristics of HIV Patients

**Figure 3:**
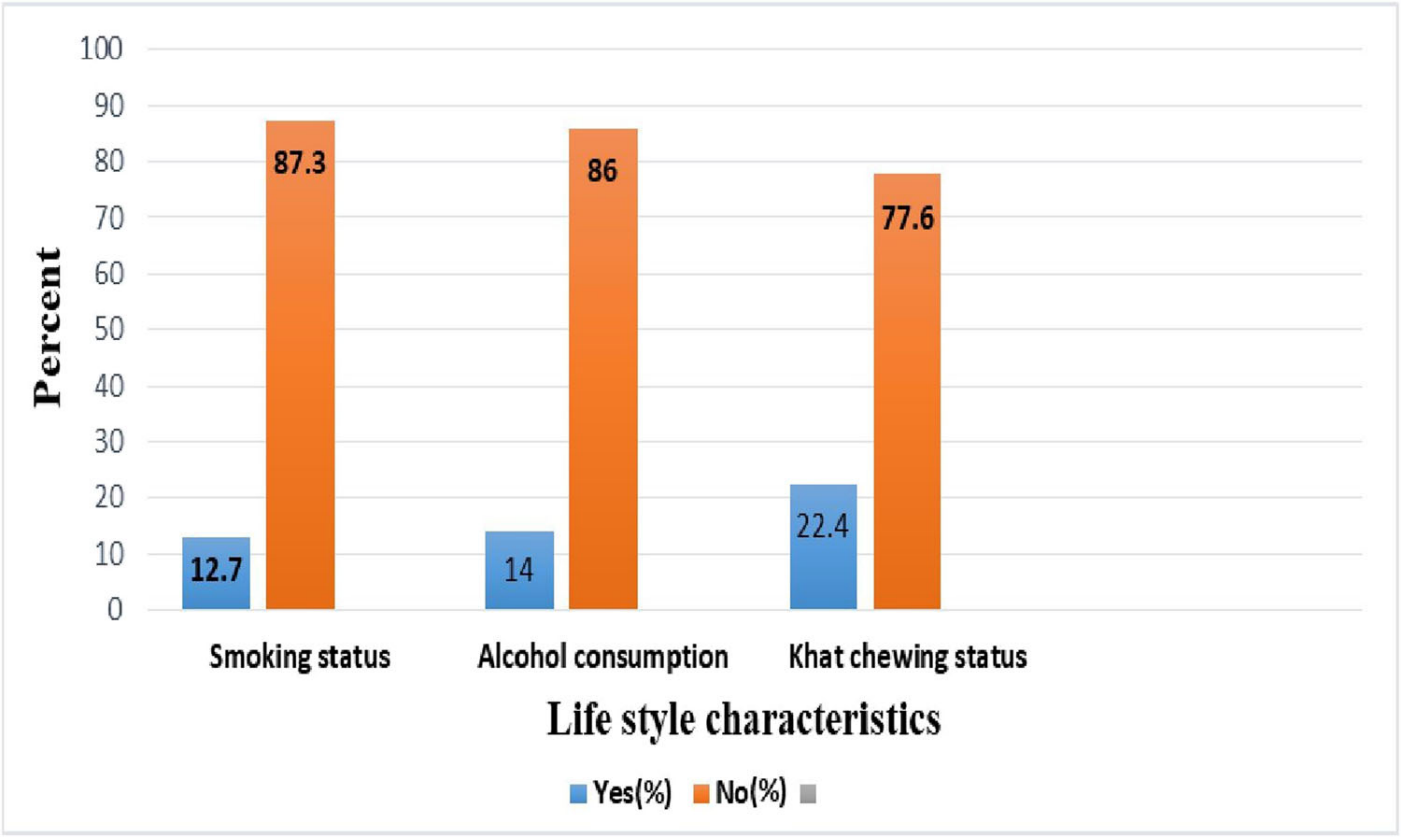
Lifestyle characteristics of HIV patients on HAART at HFCSH, 2024.

In the study, it was found that out of the total participants, 12.7% (29/228) were cigarette smokers. Regarding chewing khat, 22.4% (51/228) of them were khat chewers, and 14.0% (32/228) of them were alcohol drinkers (Figure 3).

### 3.3 Clinical Characteristics of HIV Patients

Among the study participants included in the study, 21.5% (49/228) had a family history of kidney disease. Regarding the HAART regimen, nearly one‐third of patients 31.6% (72/228) initially started with TDF + 3TC + EFV; however, approximately 96.1% (219/218) of the patients eventually switched to different regimen. The most common reason for switching was the availability of new drugs, 63.5% (139/228), followed by toxicity, 15.1% (33/228). Furthermore, majority of the participants had been on HAART for more than 10 years [74.1% (169/228)]. The mean (SD) body mass index of study participants was 23.5 (4.4 Kg/m^2^), and more than half of the study participants 57.0% (130/228) had a normal weight (Table 2).

**Table 2:** Clinical characteristics of HIV patients on HAART at HFCSH, Harar, Eastern Ethiopia, 2024 (*n* = 228).

Variables	Categories	Frequency (%)
Family history of kidney disease	Yes	49 (21.5)
	No	179 (78.5)
Initial HAART regimen	AZT + 3TC + EFV	31 (13.6)
	TDF + 3TC + EFV	72 (31.6)
	ABC + 3TC + EFV	29 (12.7)
	TDF + 3TC + DTG	29 (12.7)
	AZT + 3TC + NVP	45 (19.7)
	TDF + 3TC + NVP	22 (9.7)
Switching status	Yes	219 (96.1)
	No	9 (3.9)
Switching reason	Toxicity	33 (15.1)
	New drug available	139 (63.5)
	Clinical failure	19 (8.7)
	Immunological failure	28 (12.7)
Second HAART regimen	TDF + 3TC + DTG	139 (63.5)
	ABC + 3TC + ATV	21 (9.6)
	TDF + 3TC + ATV	30 (13.7)
	AZT + 3TC + DTG	29 (13.2)
Duration on HAART	1–5 years	13 (5.7)
	6–10 years	46 (20.2)
	More than 10 years	169 (74.1)
Drug taken	Trimethoprim/sulfamethoxazole	28 (12.3)
	Acyclovir	3 (1.3)
	Nonsteroidal anti‐inflammatory drugs (NSAIDs) (e.g., ibuprofen, naproxen)	30 (13.2)
	Combination of drugs	167 (73.2)
TB infection after HIV diagnosed	Yes	39 (17.1)
	No	189 (82.9)
BMI (mean ± SD)	23.5 ± 4.4	
	Normal	130 (57.0)
	Underweight	26 (11.4)
	Overweight	42 (18.4)
	Obese	30 (13.2)
WHO stage	Stage 1	202 (88.6)
	Stage 2	5 (2.2)
	Stage 3	18 (7.9)
	Stage 4	3 (1.3)
CD4 count (cell/mm^3^)	≤ 199	21 (9.2)
	200–349	29 (12.7)
	350–499	66 (29.0)
	≥ 500	112 (49.1)
Viral load (copies/mL)	Undetected	201 (88.2)
	< 1000	19 (8.3)
	≥ 1000	8 (3.5)

Abbreviations: ABC, abacavir; ATV, atazanavir; AZT, azidothymidine; CD4 count, cluster of differentiation 4 count; DTG, dolutegravir; EFV, efavirenz; HAART, highly active antiretroviral therapy; HIV, human immunodeficiency virus; NVP, nevirapine; SD, standard deviation; TB, tuberculosis; 3TC, lamivudine; TDF, tenofovir disoproxil; WHO, World Health Organization.

### 3.4 Prevalence and Stages of CKD

**Figure 4:**
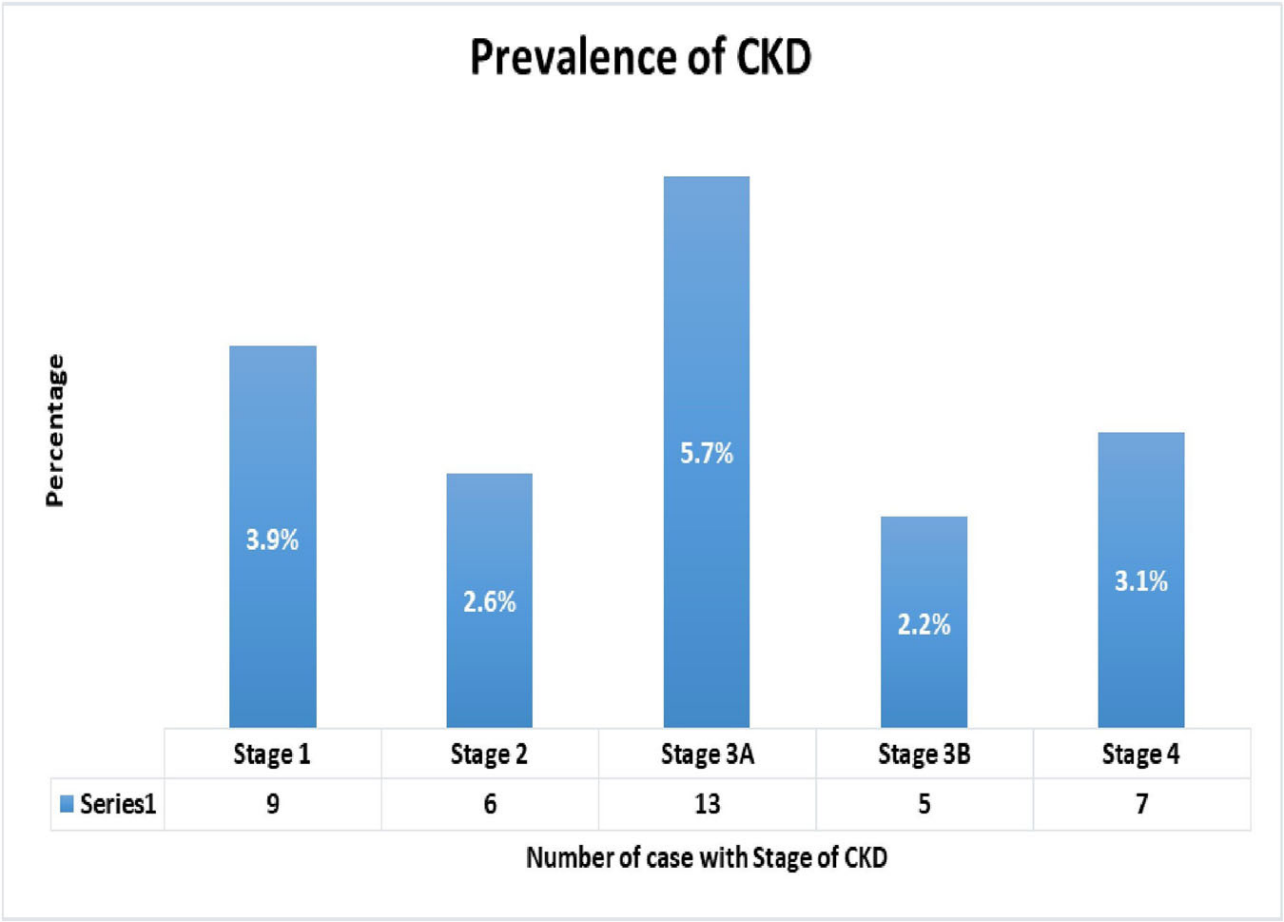
Stage of chronic kidney disease using CKD‐EPI method among HIV patients on HAART at HFCSH, Harar, Eastern Ethiopia, 2024 (*n* = 228).

According to the CKD‐EPI method, the overall prevalence of CKD was 17.5% (40/228) (95% CI: 12.8%–23.1%). Out of this, 3.9% (9/228) of the participants were categorized as Stage 1, 2.6% (6/228) were Stage 2, and 5.7% (13/228) were Stage 3A (Figure 4).

### 3.5 Factors Associated With CKD

In the bivariate logistic regression analysis, factors such as age, smoking, family history of kidney disease, body mass index, and CD4 count demonstrated a *p*‐value of less than 0.2. However, in the multivariable logistic regression analysis, only age and family history of kidney disease, were significantly (*p* < 0.05) associated with CKD (Table 3).

**Table 3:** Multivariable analysis for factors associated with chronic kidney disease among HIV patients receiving HAART at HFCSH, Harar, Eastern Ethiopia, 2024 (*n* = 228).

Variables		CKD		AOR (95% CI)	*p* value
		Yes (%)	No (%)		
Age	15–29	5 (15.2)	28 (84.8)	1	
	30–39	5 (10.2)	44 (89.8)	0.68 (0.17–2.81)	0.598
	40–49	7 (7.6)	85 (92.4)	0.39 (0.10–1.51)	0.175
	≥ 50	23 (42.6)	31 (57.4)	3.60 (1.08–11.96)	0.036*
Smoking status	Yes	8 (27.6)	21 (72.4)	2.32 (0.81–6.66)	0.119
	No	32 (16.1)	167 (83.9)	1	
Family history of kidney disease	Yes	18 (36.7)	31 (63.3)	2.99 (1.27–7.04)	0.012*
	No	22 (12.3)	157 (87.7)	1	
Body mass index	Normal	20 (15.4)	110 (84.6)	1	
	Underweight	4 (15.4)	22 (84.6)	0.54 (0.13–2.20)	0.392
	Over weight	6 (14.3)	36 (85.7)	0.99 (0.32–3.07)	0.998
	Obese	10 (33.3)	20 (66.7)	2.14 (0.73–6.26)	0.167
CD4 count	≤ 199	7 (33.3)	14 (66.7)	2.77 (0.77–9.92)	0.118
	200–349	7 (24.1)	22 (75.9)	2.09 (0.64–6.79)	0.220
	350–499	10 (15.2)	56 (84.8)	0.97 (0.37–2.57)	0.960
	≥ 500	16 (14.3)	96 (85.7)	1	

*Note:* * = Statistically significant association.

Abbreviations: AOR, adjusted odds ratio; CI, confidence interval; CKD, chronic kidney disease; CD4 count, cluster of differentiation 4 count.

## 4 Discussion

This study aimed to determine the prevalence of CKD and its associated factors among HIV patients receiving HAART at HFCSH. The results indicated an overall CKD prevalence of 17.5% (40/228) (95% CI: 12.8%–23.1%). Notably, older age (over 50 years) and a family history of kidney disease were significantly associated with CKD in this population.

The prevalence observed in this study was higher compared to studies conducted in Taiwan 7.03% [22], Mexico 11.7% [23], South Africa 7.0% [24], Ivory Coast 10.4% [12], Nigeria 6.5% [25], Ghana 12.6% [26], and Ethiopia (Jimma 7.6% [15]). This difference might be due to the fact that most of the study participants, 78.1% (178/228), in this study received TDF based HAART regimen. Previous studies have indicated that TDF drug is known to decrease estimated GFR [27, 28], which could potentially contribute to the higher prevalence of CKD observed in this study. Additionally, participants' lifestyles and the criteria used for diagnosing CKD may also account for these differences.

In contrast, the prevalence of CKD in this study was lower than that found in studies from France 39.0% [13], Burundi 45.7% [29], and Cameroon 26.5% [30]. This variation might be due to the inclusion of known CKD risk factors, such as diabetes and hypertensive patients, in the French and Cameroonian studies, which could result in a higher prevalence. Furthermore, differences in diagnostic criteria and the equations used for estimating GFR may have influenced the results.

The prevalence of this study was consistent with previous studies conducted in Nigeria 15.3% [16], Malawi 22.4% [31], Uganda 14.4% [32], and Ethiopia (Gondar 16.1% [6], Bahir Dar 12.9% [14], and Mettu 20.7% [11]).

Our finding revealed that older age (≥ 50 years) [AOR: 3.6, 95% CI: 1.08–11.96, *p* = 0.036] was significantly associated with CKD among HIV‐positive patients on HAART. The odds of developing CKD were 3.6 times higher for individuals over 50 compared to those aged 15–29 years. This finding is consistent with studies conducted in China [33], Mexico [23], France [13], Ivory Coast [12], and Tanzania [34].

The observed association may be attributed to several factors related to both structural and functional changes in the kidneys. First, as age increases, the number of nephrons gradually declines. This decrease is influenced by various factors, including oxidative stress and inflammation, which ultimately result in reduced overall filtration capacity and a lower GFR. Additionally, structural changes occur in the glomeruli; with aging, these structures undergo thickening and sclerosis, impairing their filtration efficiency and contributing to a further decline in GFR [35, 36, 37]. Moreover, age‐related vascular changes—such as the thickening and stiffening of blood vessels, reduced blood flow, and impaired regulation of renal blood flow can restrict blood delivery to the kidneys, compromising their function and further decreasing GFR [38].

A family history of kidney disease [AOR: 2.9, 95% CI: 1.27–7.04, *p* = 0.012] was another significant factor associated with CKD among HIV patients on HAART. Those with a family history were 2.9 times more likely to develop CKD than those without such a history. This association is supported by research indicating that individuals with a first‐degree relative affected by CKD face a significantly higher risk of developing the disease [39]. Shared environmental factors, such as lifestyle, also play a role; families often have similar dietary habits (including high salt and fat intake), levels of physical activity, and smoking behaviors, all of which can contribute to comorbidities that increase the risk of CKD [40].

The strengths of this study were that CKD was classified based on both proteinuria and eGFR, and assessments were made for both initial and current HAART regimens taken by HIV patients. Additionally, the recent and recommended CKD‐EPI 2021 equation was used to calculate eGFR. This study also has some limitations. The first limitation was that it was a cross‐sectional study, which cannot show the cause‐and‐effect relationship between CKD and independent variables. The second limitation was that the sample size was relatively small, which could limit the generalizability of the findings to a larger population.

## 5 Conclusion and Recommendations

CKD was diagnosed by evaluating proteinuria and eGFR, which was calculated using CKD‐EPI 2021 equation. Based on these two parameters, this study showed an overall prevalence of 17.5%. According to the findings, approximately one in six HIV patients receiving HAART were diagnosed with CKD. The analysis also identified older age and having a family history of kidney disease as significant factors associated with CKD.

Regular monitoring of kidney function (at least every 6 months) is important for older individuals and those with a family history of kidney disease to promptly identify and effectively manage CKD in individuals on HAART. In high‐burden areas, training healthcare providers and launching public health campaigns to raise awareness about CKD are crucial steps toward improving patient outcomes. Additionally, we recommend that policymakers integrate kidney health into HIV care programs to ensure routine kidney function monitoring becomes a standard part of HIV treatment. Furthermore, longitudinal studies with larger sample sizes are needed to explore additional factors contributing to CKD development in this population.

## Author Contributions

**Gada Diba:** conceptualization, investigation, writing – original draft, methodology, writing – review and editing, software, formal analysis, project administration, data curation, funding acquisition, resources. **Ahmedmenewer Abdu:** conceptualization, investigation, methodology, visualization, validation, writing – review and editing, software, formal analysis, supervision. **Ephrem Tefera Solomon:** conceptualization, investigation, methodology, validation, visualization, writing – review and editing, software, formal analysis, supervision. **Winner Kucha:** conceptualization, investigation, methodology, validation, visualization; writing – review and editing, software, formal analysis, supervision.

## Ethics Statement

Ethical clearance was obtained from Institutional Health Research Ethical Review Committee (Ref No: IHRERC/215/2023) belonging to College of Health and Medical Sciences, Haramaya University. Written informed consent was obtained from each study participant after providing a detailed explanation of the research. The study was conducted in accordance with the Helsinki Declaration.

## Consent

The authors have nothing to report.

## Conflicts of Interest

The authors declare no conflicts of interest.

## Transparency Statement

The lead author Gada Diba affirms that this manuscript is an honest, accurate, and transparent account of the study being reported; that no important aspects of the study have been omitted; and that any discrepancies from the study as planned (and, if relevant, registered) have been explained.

## Data Availability

The data that support the findings of this study are available from the corresponding author upon reasonable request.
